# Cross-continental phylogeography of two Holarctic Nymphalid butterflies, *Boloria eunomia* and *Boloria selene*

**DOI:** 10.1371/journal.pone.0214483

**Published:** 2019-03-26

**Authors:** Jana Maresova, Jan Christian Habel, Gabriel Neve, Marcin Sielezniew, Alena Bartonova, Agata Kostro-Ambroziak, Zdenek Faltynek Fric

**Affiliations:** 1 Biology Centre of the Czech Academy of Sciences, Institute of Entomology, Ceske Budejovice, Czech Republic; 2 University of South Bohemia, Faculty of Sciences, Ceske Budejovice, Czech Republic; 3 Terrestrial Ecology Research Group, Department of Ecology and Ecosystem Management, School of Life Sciences Weihenstephan, Technische Universität München, Freising, Germany; 4 IMBE, Aix Marseille Université, CNRS, IRD, Avignon Université, Marseille, France; 5 Laboratory of Insect Evolutionary Biology and Ecology, Institute of Biology, University of Białystok, Białystok, Poland; Institute of Systematics and Evolution of Animals Polish Academy of Sciences, POLAND

## Abstract

Pleistocene glaciations had significant effects on the distribution and evolution of species inhabiting the Holarctic region. Phylogeographic studies concerning the entire region are still rare. Here, we compared global phylogeographic patterns of one boreo-montane and one boreo-temperate butterflies with largely overlapping distribution ranges across the Northern Hemisphere, but with different levels of range fragmentation and food specialization. We reconstructed the global phylogeographic history of the boreo-montane specialist *Boloria eunomia* (n = 223) and of the boreo-temperate generalist *Boloria selene* (n = 106) based on mitochondrial and nuclear DNA markers, and with species distribution modelling (SDM). According to the genetic structures obtained, both species show a Siberian origin and considerable split among populations from Nearctic and Palaearctic regions. According to SDMs and molecular data, both butterflies could inhabit vast areas during the moderate glacials. In the case of *B*. *selene*, high haplotype diversity and low geographic structure suggest long-lasting interconnected gene flow among populations. A stronger geographic structuring between populations was identified in the specialist *B*. *eunomia*, presumably due to the less widespread, heterogeneously distributed food resources, associated with cooler and more humid climatic conditions. Populations of both species show opposite patterns across major parts of North America and in the case of *B*. *eunomia* also across Asia. Our data underline the relevance to cover entire distribution ranges to reconstruct the correct phylogeographic history of species.

## Introduction

The Quaternary period is characterized by severe fluctuations of long glacial and shorter interglacial periods. These climatic changes caused repeated latitudinal expansions and retractions of species´ distributions [1,2]. According to the “classical” refugia scenario, thermophilic species survived glacial periods in geographically distinct southern refugia, disappeared from major parts of their northerly distribution, and re-expanded again in the wake of postglacial warming [3,4]. Cold-adapted species reacted contrary, with shifts into higher latitudes and altitudes during the warmer interglacial stages [5,6]. However, a number of recent studies showed that the situation is much more complex, emphasizing the importance of the extra-Mediterranean refugia for both temperate and cold-adapted species [7].

Generally, cold-adapted species presently inhabit disjunct areas, consisting of a rather continuous circumpolar distribution and isolated patches restricted to higher elevations in southern mountains [8]. Depending on specific habitat association, arctic-alpine species would inhabit open northern treeless tundras plus mountains above the tree line, while boreo-montane species would occur in northern and mountain taigas and in bogs [8]. For all the northern species, Schmitt [9] proposed two biogeographic scenarios: 1) interglacial population isolation persisted during glacial eras, resulting in deeper genetic splits; or 2) continuous distribution throughout the ice ages with subsequent gene flow, interrupted in the wake of postglacial warming, causing rather weak genetic differentiation.

Although both arctic-alpine and boreo-montane species ranges encompass large parts of the Holarctic region [8], studies on the latter are still underrepresented in the literature on the biogeography of biota during the Pleistocene. Few boreo-montane taxa were studied in the western Palaearctic region [10–15], neglecting the fact that most of them occur across the Northern Hemisphere. Considering the vast extent and largely ice-free status of north-central Asia and north-western North America during the Quaternary, these areas must have played an important role in structuring high-latitude biodiversity and boreo-montane species [16–18]. For instance, the Beringian region might have served as northern refugium for many boreal plant and animal species [19,20]. However, only few studies on the phylogeography of boreo-montane species, including entire distribution ranges, have been published so far (cf. [21–23]).

In this study, we contribute to the knowledge of the phylogeography of two species considering their entire distribution ranges across the Northern Hemisphere. We compare the genetic structures of two Holarctic butterflies, the boreo-montane Bog Fritillary *Boloria eunomia* (Esper, 1800) and the boreo-temperate Small Pearl-bordered Fritillary *Boloria selene* (Denis & Schiffermüller, 1775). Both species show similar distribution ranges, including Asia, Europe and North America [24–26], but the two model taxa differ in respect of their habitat demands and ecological specialisation: *Boloria eunomia* inhabits bogs and fens across the Holarctic region, syntopically with species such as *Colias palaeno* (Linnaeus, 1761) and *Oeneis jutta* (Hübner, [1805]) [27]. *Boloria eunomia* shows a disjunct distribution across central and southern Europe and occur restricted to wet meadows and bogs at higher altitudes, with high abundances of the single larval host plants *Polygonum bistorta* L. and *P*. *viviparum* L. [28,29]. This butterfly is threatened due to the habitat loss or incorrect habitat management across central Europe [30], but may swiftly enlarge its distribution when the habitats are suitable [15,25]. Towards North and East, *B*. *eunomia* becomes more broadly distributed and common, as it inhabits extensive habitats, which are both moist tundra and willow seeps as well as raised peat bogs. There, *Viola palustris* L. and *Oxycoccus palustris* Pers. are known as additional food plants [25,31,32]. In contrast, the more generalist *B*. *selene* uses a broader variety of different habitats such as forest clearings, wet meadows, bogs and marshy areas near lakes. The latter species is distributed across the entire Holarctic region, but in contrast to *B*. *eunomia*, it does not reach the Siberian arctic and it occurs also at lower altitudes and latitudes [33]. Larval food plants are several *Viola* spp. [34–36].

In our study, we applied molecular analyses (mitochondrial and nuclear genes) and performed species distribution models (SDMs) to reconstruct the phylogeography of these butterflies. We hypothesize that *B*. *eunomia*, inhabiting bogs and humid meadows, represent a strong genetic differentiation due to its rather patchy occurrence during glacial and interglacial stages. On the other hand, we assume that the *B*. *selene* populations are not or only marginally genetically differentiated among each other as this species probably occupied the same open habitats during both eras, and thus population disjunction occurred only recently.

## Material and methods

### Sampling

We sampled 124 individuals of *Boloria eunomia* and 60 individuals of *B*. *selene*, covering the whole Holarctic region. The samples were obtained by the authors and from private and public museum collections. A further 99 sequences of *B*. *eunomia* and 46 of *B*. *selene* were added to the dataset from GenBank (http://www.ncbi.nlm.nih.gov/) (S1 Table, including specimen ID numbers, GenBank accession numbers, information about specimen repository).

### Acquisition of genetic data

DNA was extracted from legs or the abdomen using the Genomic DNA Mini Kit—Tissue (Geneaid) following the manufacturer’s protocols. One fast mitochondrial (*cytochrome c oxidase subunit* I, COI) and two more conservative nuclear (*arginine kinase*, ArgKin; *wingless*, WG) genes were amplified by Polymerase Chain Reactions (PCR) in 20 μl volume (10 μl PPP Mastermix Top-Bio, 6.9 μl PCR H2O, 0.8 + 0.8 μl primers, 1.5 μl DNA). Two forward-reverse primer pairs were used for COI: LCO/HCO and Ron/HCO; for ArgKin, one pair: ArginineF/ArginineR; and for *wingless*, one pair: LepWG1/LepWG2 [37,38]. The universal tails T7Promoter and T3 were attached to all primers. The thermal cycling profile was 95 °C for 5 min, 94 °C for 30 s, 50 °C for 30 s, 72 °C for 90 s, for 36 cycles, and final extension 72 °C for 10 min. Sequencing was provided by Macrogen Inc. on ABI3730XL DNA analysers. Sequences were checked manually and aligned in Geneious v. 8.0.5 [39].

### Genetic diversity and population structure

Maximum parsimony haplotype networks were reconstructed to illustrate relationships among haplotypes of *B*. *eunomia* and *B*. *selene* using TCS algorithm [40] in PopArt [41]. The population structure was evaluated by spatial analysis of molecular variance (SAMOVA) implemented in SPADS 1.0 [42], with 10,000 simulated annealing steps and 10 repetitions. This analysis allows defining groups of populations without a priori information on population structure. The method uses a simulated annealing algorithm that maximizes the proportion of genetic variance (Φ_CT_) partitioned among geographically adjacent populations. SAMOVA was performed for each possible number of population clusters (K) from 2 to 20, with Φ_CT_ calculated for each value of K. To calculate descriptive statistics of population groups defined by SAMOVA, we used Arlequin 3.5 [43,44].

### Tree construction and time estimates

The best substitution models were selected using PartitionFinder 1.1.1 (see Table 1) [45]. Phylogeny and time diversification of *Boloria eunomia* (COI, ArgKin) and *B*. *selene* (COI) haplotypes were estimated in BEAST 1.8.4 [46]. We used an uncorrelated lognormal relaxed clock with a coalescent constant size tree and normal distribution priors. For model calibration, we used reference points from the family Nymphalidae [47,48] and the genus *Boloria* [49]; fossils of *Boloria* are unknown. We used three calibration points in *B*. *eunomia*: first, 12.8 ± 2 Mya for the split between outgroups (*B*. *aquilonaris* (Stichel, 1908), *B*. *napaea* (Hoffmannsegg, 1804), *B*. *pales* (Denis & Schiffermüller, 1775)) + *B*. *eunomia* and a common ancestor of their sister species; second, the split between outgroups and *B*. *eunomia* 10.2 ± 2.0 Mya; third, the split between populations from Eurasia and North America 2.0 ± 0.5 Mya. In *B*. *selene*, we used also three secondary calibration points: first, 9.2 ± 2.0 Mya was the split between outgroups *B*. *improba* (Butler, 1877) and *B*. *thore* (Hübner, [1803]) + *B*. *selene*; second, the split between *B*. *selene* and *B*. *thore* 5.0 ± 1.2 Mya; third, the split between populations from Eurasia and North America 2.8 ± 0.8 Mya. We specified MCMC chain length of 50,000,000 generations, sampling every 1000 steps. Operators were auto-optimised. The parameter ucld.mean was set as lognormal, while other parameters were left in default values. Three independent runs were performed on each dataset. Tracer 1.6 [50] was used to evaluate estimated values and effective sample size (ESS) for each model parameter and to check convergence. All three runs were combined, and a single best tree was identified. The trees were visualized using the software FigTree 1.3.1. We ran the analyses using the Cipres server [51].

**Table 1:** Substitution models for partitioned analyses identified by the PartitionFinder.

Partitions		PartitionFinder	BEAST
*Boloria eunomia* mtDNA (COI)			
p1	COI_pos1, COI_pos3	K81UF	GTR
p2	COI_pos2	TIM + G	GTR + G
*Boloria eunomia* nDNA (ArgKin)			
p1	ArgKin_pos1, ArgKin_pos2	F81	HKY
p2	ArgKin_pos3	TVM + G	GTR + G
*Boloria selene* mtDNA (COI)			
p1	COI_pos1, COI_pos2	HKY + I	HKY + I
p2	COI_pos3	GTR	GTR

If the model was not implemented in BEAST, the first available model with the lowest Bayesian Information Criterion (BIC) scores was used.

### Species distribution model (SDM)

To investigate climatically suitable areas for the species, we build SDM using georeferenced species records for both species from our own or our colleagues´ fieldwork, from publicly available data from the Global Biodiversity Information Facility (GBIF; http://www.gbif.org) and from the literature [52–56]. Duplicates were erased. This resulted in a total of 161 records for *B*. *eunomia* and 280 for *B*. *selene* covering the whole species ranges. We obtained information on the present climate and for the climate during the Last Glacial Maximum (LGM; 22000 years ago) as the 19 BIOCLIM variables (BIO1–BIO19) described by the community climate system model (CCSM), the Worldclim 1.4 database [57], at 2.5 arc min spatial resolution. We restricted the area to the Northern Hemisphere (35‒75°N, −180°W to 180°E). We calculated the SDMs using MaxEnt 3.4.1 [58] via the R environment using packages ‘dismo’ and ‘raster’ [59,60]. Since the records tend to be biased towards the accessible locations (cf. [61]), we used bias background correction for road density [Center for International Earth Science Information Network (CIESIN) Columbia University & Information Technology Outreach Services (ITOS) University of Georgia, 2013], which we rasterized in QGIS v.2.14.3 [62]. We tested the records against 10,355 random background points selected by the script ‘sampleRast’ (available at https://rdrr.io/github/adamlilith/enmSdm/man/sampleRast.html). We obtained the best values of the intrinsic MaxEnt estimates, regularization multiplier (RM) and the response of feature classes (FC) using the ‘ENMeval’ R package [63]. We selected the variables with lowest AICc (AIC corrected for small samples) values after K − 1 jackknife data partitioning. We measured the relative importance of the BIOCLIM variables in the MaxEnt using the jacknife test and excluded the variables with negative gain or gain close to zero expressed as the difference between full model training gain and the gain of the model without the variable(s).

## Results

ArgKin was amplified only for *B*. *eunomia* and WG only for *B*. *selene*, possibly due to mutations in the primer region. The latter did not show any intraspecific variation throughout the sampled populations (91 specimens: for GenBank accessions numbers see S1 Table). Consequently, subsequent molecular analyses were conducted only with COI for *B*. *selene* and COI and ArgKin for *B*. *eunomia*.

### *Boloria eunomia* cytochrome *c* oxidase

**Fig 1:**
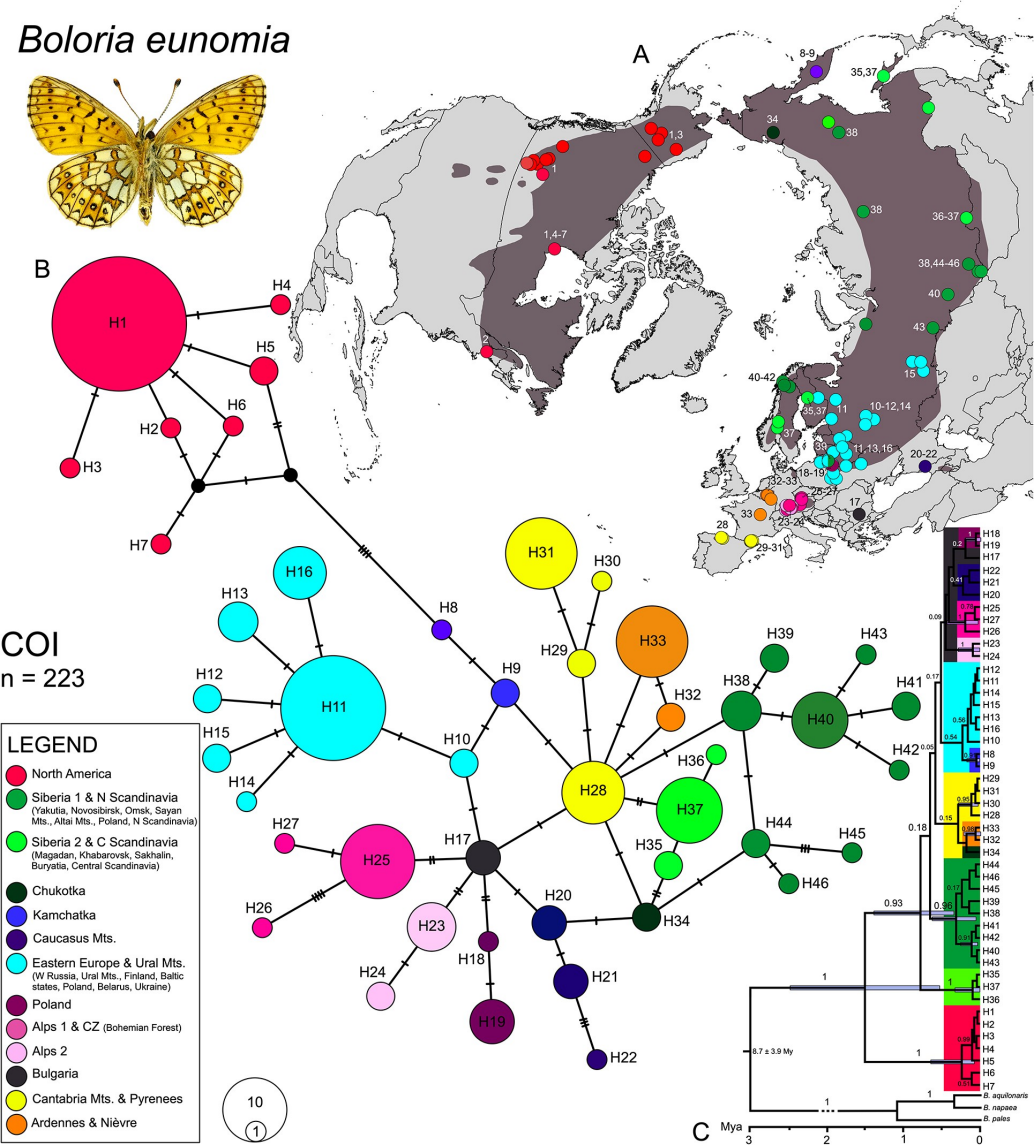
Phylogeographic patterns of *Boloria eunomia* based on the mitochondrial (COI) marker. Haplotypes distribution on the map COI (A). TCS haplotype network illustrating relationships of the 46 COI haplotypes (B); mutations are shown as hatch marks; the colours correspond to the map (A). Chronogram calculated in BEAST based on COI (C); node bars display 95% highest posterior density interval of the molecular clock and branch labels show posterior probabilities.

We obtained 670 bp COI sequences for 223 individuals from 95 localities and identified 46 haplotypes (Fig 1A and 1B). According to the haplotype network (Fig 1B), haplotypes from Canada and USA (H1-7) formed a separate group differentiated from the Palaearctic, characterised by a widespread haplotype (H1) occurring in high frequencies. Within the Palaearctic region, relatively high haplotype diversity was observed, the highest across Russia with affinities to Scandinavia (H8-16, H20-22, H34-46). European populations were considerably differentiated (H17-19, H23-33). The populations from Cantabrian Mts. (H28) and the Pyrenees (H29-30) slightly differed from each other and were simultaneously related to populations from Ardennes and Nièvre (H32-33). Within Central Europe, two lineages were detected in the Alps: H23-24 and H25-27, the latter closely related to the population from the Czech Republic (the Bohemian Forest; Fig 1 and Figure A in S1 Fig). Poland might represent a contact zone for different lineages (H10-16, H18-19 and H39; Fig 1 and Figure B in S1 Fig). Populations from Eastern Europe (H10-14, H16, H18-19), Balkan region (H17) and Ural Mts. (H15) are closely related (Fig 1).

**Fig 2:**
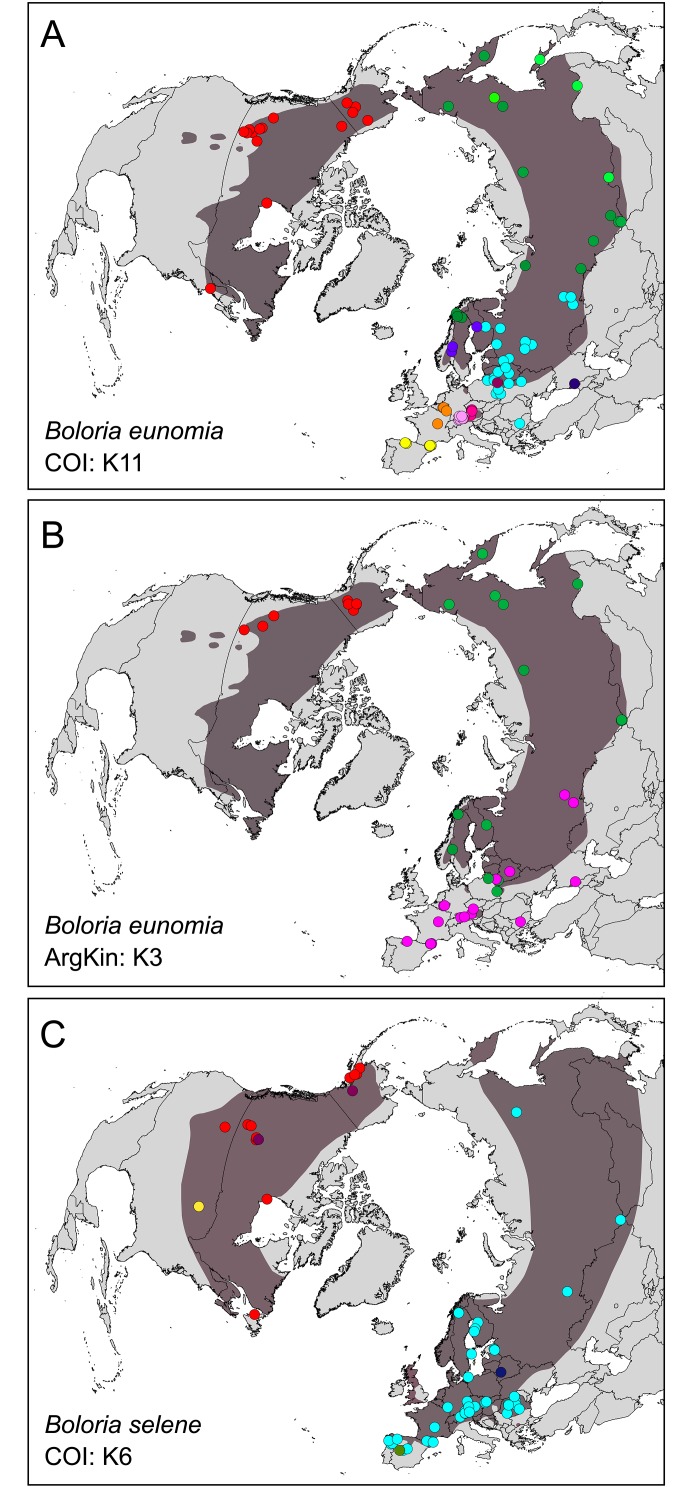
Spatial analyses of molecular variance (SAMOVA) displayed on the maps. Eleven mitochondrial (COI) lineages of *Boloria eunomia* (A); three nuclear (ArgKin) lineages of *Boloria eunomia* (B); six mitochondrial (COI) lineages revealed in *Boloria selene* (C).

In SAMOVA, the largest decrease of Φ_CT_ variance was for *K* = 11 (Φ_CT_ = 0.77), distinguishing 11 population groups: (1) A1-CZ: Alps 1 & CZ; (2) A2: Alps 2; (3) CA-PY: Cantabria Mts. & Pyrenees; (4) AR-NI: Ardennes & Nièvre; (5) EE-UR: Eastern Europe & Ural Mts.; (6) CS: Central Scandinavia; (7) NS-SIB1: Northern Scandinavia & Siberia 1; (8) PL: Poland; (9) SIB2: Siberia 2; (10) CAU: Caucasus Mts. and (11) NA: North America (Fig 2A and Figure A in S2 Fig). The total haplotype diversity was high (Hd = 0.92), with the highest values for NS-SIB1 (Hd = 0.87), SIB2 (Hd = 0.83) and CAU (Hd = 0.71). Significant negative values of Tajima’s D for A1-CZ and NA and Fu’s *F*s for EE-UR, NS-SIB1 and NA indicate recent population expansion, genetic hitchhiking or selection [64,65] (see Table 2).

**Table 2:** Summary of genetic diversity indices and neutrality tests for *Boloria eunomia* (COI, ArgKin) and *Boloria selene* (COI).

Group		N	S	h	Haplotypes	Hd ± SD	π ± SD	Tajima’s D	Fu’s FS
*Boloria eunomia* mtDNA (COI)									
1	Alps 1 & CZ (A1-CZ)	14	6	3	H25—H27	0.2747 ± 0.1484	0.0013 ± 0.0011	- 1.9589**	0.5351
2	Alps 2 (A2)	10	6	3	H23, H24	0.6222 ± 0.1383	0.0035 ± 0.0023	0.3710	0.5351
3	Cantabria Mts. & Pyrenees (CA-PY)	25	3	4	H28—H31	0.6167 ± 0.0639	0.0016 ± 0.0012	0.8924	0.4115
4	Ardennes & Nièvre (AR-NI)	14	1	2	H32, H33	0.2637 ± 0.1360	0.0004 ± 0.0005	- 0.3414	0.1857
5	Eastern Europe & Ural Mts. (EE-UR)	50	10	9	H10—H17, H39	0.6686 ± 0.0706	0.0018 ± 0.0013	- 1.2880	- 3.1749*
6	Central Scandinavia (CS)	10	1	2	H35, H37	0.2000 ± 0.1541	0.0003 ± 0.0005	- 1.1117	- 0.3393
7	N Scandinavia & Siberia 1 (NS-SIB1)	25	12	11	H8, H9, H34, H38, H40—H46	0.8733 ± 0.0496	0.0039 ± 0.0024	- 0.6109	- 3.5831*
8	Poland (PL)	6	1	2	H18, H19	0.3333 ± 0.2152	0.0005 ± 0.0007	- 0.9330	- 0.0028
9	Siberia 2 (SIB2)	4	2	3	H35—H37	0.8333 ± 0.2224	0.0015 ± 0.0015	- 0.7099	- 0.8873
10	Caucasus Mts. (CAU)	7	6	3	H20—H22	0.7143 ± 0.1267	0.0032 ± 0.0023	- 0.6351	1.4625
11	North America (NA)	58	6	7	H1—H7	0.2281 ± 0.0734	0.0005 ± 0.0005	- 1.8875**	- 6.7157**
	Total	223	47	46	H1—H46	0.9159 ± 0.0114	0.0098 ± 0.0052	- 0.4794	- 13.864**
*Boloria eunomia* nDNA (ArgKin)									
1	W Palaearctic (WP)	38	2	4	H4—H7	0.4737 ± 0.0728	0.0009 ± 0.0009	0.2595	- 0.8485
2	Scandinavia & Siberia (SC-SIB)	22	18	14	H8—H21	0.9177 ± 0.0455	0.0057 ± 0.0034	- 1.1663	- 6.7518**
3	North America (NA)	17	5	3	H1—H3	0.2279 ± 0.1295	0.0010 ± 0.0010	- 1.9433**	- 0.0292
	Total	77	30	21	H1—H21	0.8312 ± 0.0299	0.0085 ± 0.0046	- 0.5251	- 3.4341
*Boloria selene* mtDNA (COI)									
1	Eurasia (E)	82	52	25	H9—H19, H21—H34	0.7064 ± 0.0560	0.0042 ± 0.0026	- 2.4878**	- 16.084**
2	Belarus (BR)	1	0	1	H20	n.a.	n.a.	n.a.	n.a.
3	Spain (SP)	1	0	1	H18	n.a.	n.a.	n.a.	n.a.
4	North America (NA)	17	4	4	H1—H4	0.5000 ± 0.1355	0.0015 ± 0.0012	- 0.8161	- 0.5062
5	Alberta (AB)	2	1	2	H5, H6	1.0000 ± 0.5000	0.0017 ± 0.0024	0.0001	0.0001
6	Wisconsin (W)	3	1	2	H7, H8	0.6667 ± 0.3143	0.0012 ± 0.0014	0.0001	0.2007
	Total	106	84	34	H1—H34	0.8045 ± 0.0376	0.0145 ± 0.0075	- 1.4995*	- 5.9885

Population groups within their Holarctic range are based on SAMOVA analyses.

(N) number of individuals sampled, (S) number of polymorphic sites, (h) number of haplotypes, (Hd) haplotype diversity, (π) nucleotide diversity, with standard deviation (SD), (D) and (Fs) statistic for neutrality test,

** p < 0.01,

* p < 0.05

The first split of the monophyletic *B*. *eunomia* clade based on BEAST estimate (Fig 1C) occurred between the Nearctic and Palaearctic haplotypes during the Pleistocene (~1.6 Mya), making the North American clade sister to all Eurasian populations. The first split within the Palaearctic region took place ~0.8 Mya, dividing the haplotypes from Central Scandinavia, Russian Far East and Sakhalin regions from the rest. The remaining Eurasian haplotypes further split into two main branches ~0.6 Mya: One branch with haplotypes from Northern Scandinavia, Poland, Omsk, Altai and Sayan Mts., Novosibirsk and Yakutia; the second with haplotypes distributed mostly in Europe (Spain, Andorra, France, Belgium, Alps, Czech Republic, Bulgaria, Poland, Belarus, Ukraine, Baltic States, S Finland, W Russia) and the Caucasus Mts., Ural Mts., Kamchatka and Chukotka. These haplotypes differentiated around ~0.5 Mya, however, relationships among them have to be considered with caution because of the weak branch support.

### *Boloria eunomia* arginine kinase

**Fig 3:**
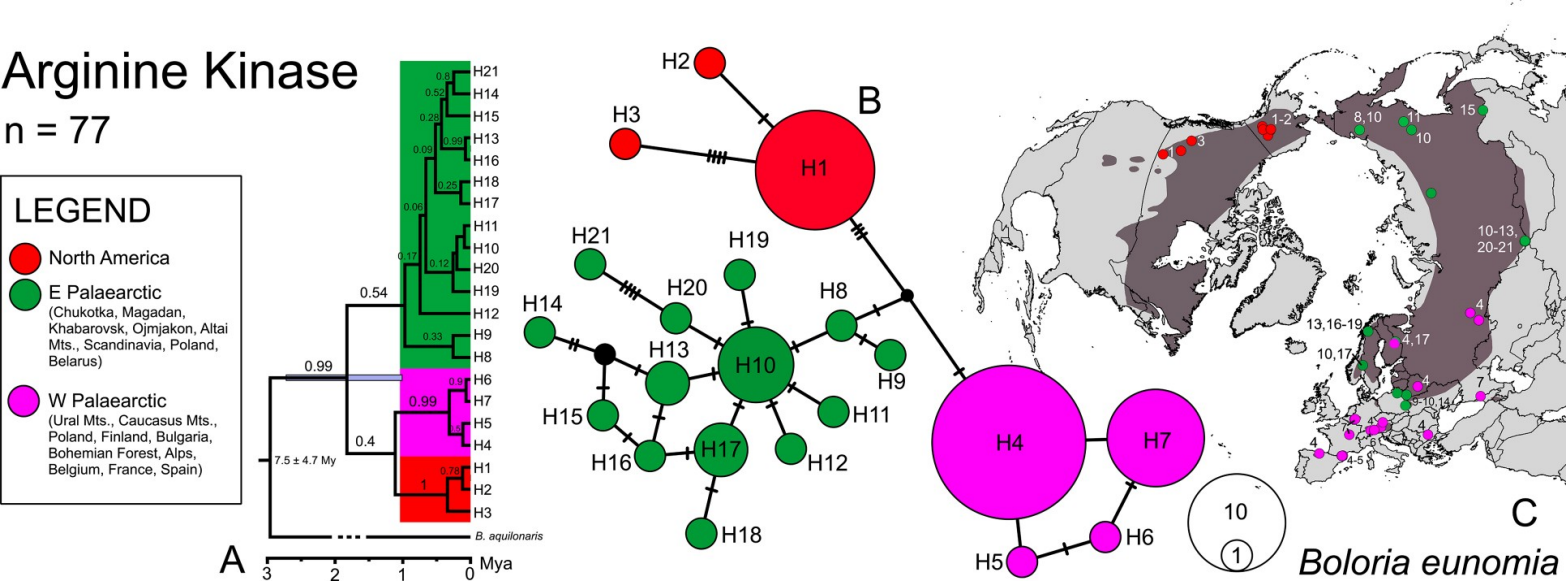
Phylogeographic patterns of *Boloria eunomia* based on nuclear (ArgKin) marker. Chronogram calculated in BEAST (A); node bars display 95% highest posterior density interval of the molecular clock and branch labels show posterior probabilities. TCS haplotype network illustrating relationships of the 21 ArgKin haplotypes (B); mutations are shown as hatch marks; the colours correspond to the haplotype distribution map (C).

We obtained 577 bp ArgKin sequences (77 individuals from 47 localities, 21 haplotypes; Fig 3A–3C). The haplotypes from the Nearctic region (H1-3) were again separated from populations of Eurasia. This nuclear marker also showed a strong relation of Scandinavian and Siberian haplotypes (H8-21). However, the general phylogeographic structuring across the Palaearctic region was weaker when compared to COI. In SAMOVA, the highest variance increase of Φ_CT_ was for *K* = 3 (Φ_CT_ = 0.65), separating: (1) NA: North America; (2) SC-SIB: Scandinavia + Poland + Siberia and (3) WP: Western Palaearctic (Spain, France, Belgium, Alps, CZ, Poland, Belarus, S Finland, Bulgaria, Caucasus and Ural Mts.) (Fig 2B and Figure B in S2 Fig). Overall haplotype diversity was again high (Hd = 0.83) with the highest value for the SC-SIB group (Hd = 0.92). Significant negative values of Tajima’s D for NA and Fu’s *F*s for SC-SIB were observed (Table 2). The BEAST tree topology (Fig 3A) was consistent with both haplotype network (Fig 3B) and SAMOVA (Fig 2B), dividing haplotypes into three groups.

### *Boloria selene* cytochrome *c* oxidase

**Fig 4:**
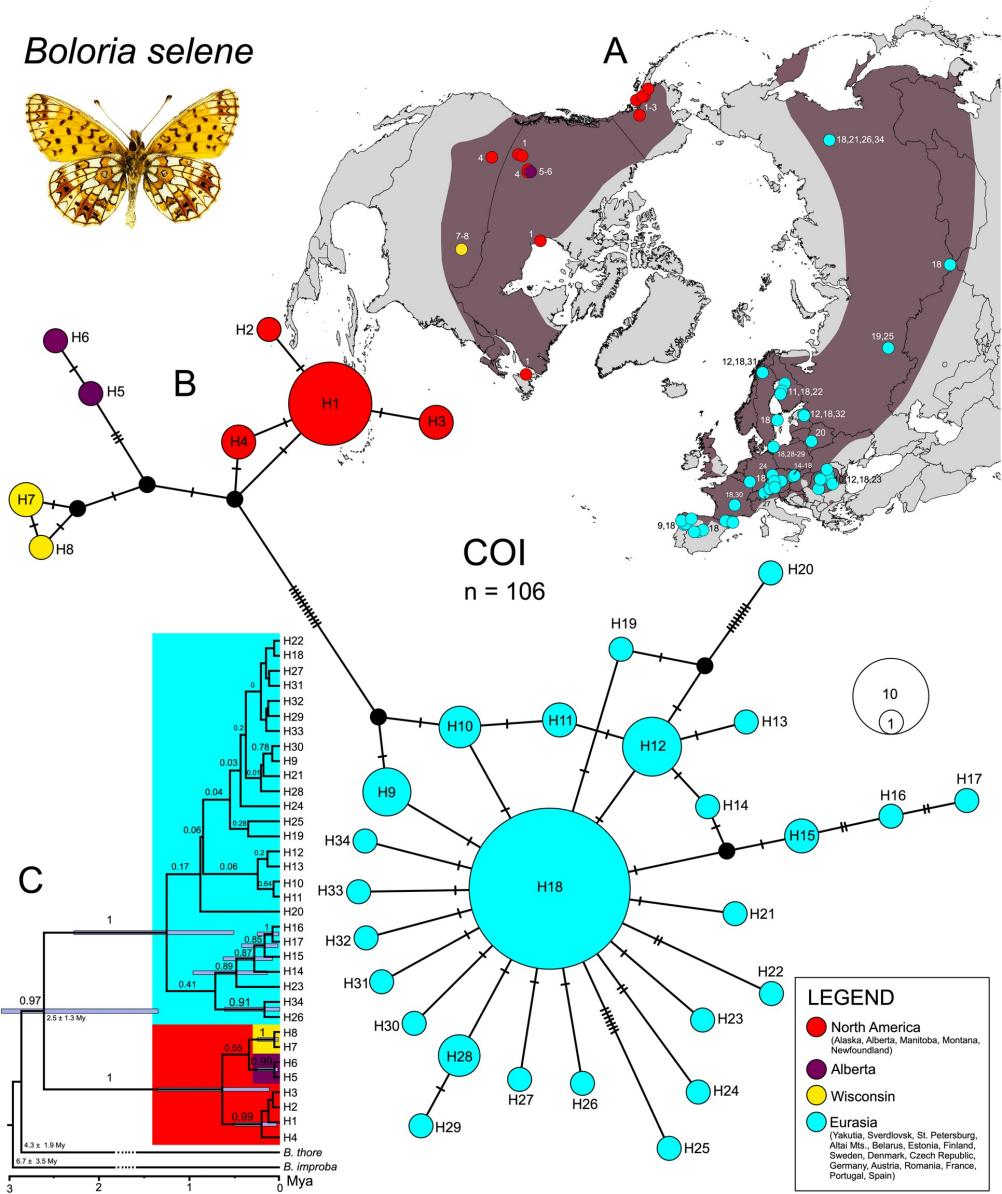
Phylogeographic patterns of *Boloria selene* based on mitochondrial (COI) marker. Haplotypes distribution on the map (A). TCS haplotype network illustrating relationships of the 34 (COI) haplotypes (B); mutations are shown as hatch marks; the colours correspond to the map. Chronogram calculated in BEAST (C); node bars display 95% highest posterior density interval of the molecular clock and branch labels show posterior probabilities.

In *B*. *selene*, we obtained 582 bp COI sequences for 106 individuals from 62 localities and identified 34 haplotypes (Fig 4A and 4B). Haplotype network showed considerable difference between haplotypes from Nearctic and Palaearctic regions (Fig 4B). Haplotypes within the North America were divided into three groups; the first one (H1-4) was characterised by one widespread haplotype occurring throughout Nearctic (H1), the second included samples from Alberta (H5-6) and the last involved Wisconsin haplotypes (H7-8). In contrast, no clear geographic pattern was visible across the Palaearctic region, characterised by star-like network structure (H9-34). In SAMOVA, large increases in Φ_CT_ occurred at K = 4, 6, 11 and 15 (Figure C in S2 Fig). We evaluated as the best K = 6 (Φ_CT_ = 0.82) because higher levels of K become less biologically informative (i.e. clusters formed by a single sample). Subsequent population grouping is depicted in Fig 2C: (1) E: Eurasia; (2) BR: Belarus; (3) SP: Spain; (4) NA: North America; (5) W: Wisconsin; (6) AB: Alberta. Total haplotype diversity was again relatively high (Hd = 0.81). In this region, significant negative values in both Tajima’s *D* and Fu’s *F*s were observed (Table 2).

According to the BEAST chronogram (Fig 4C), COI haplotypes of *B*. *selene* showed strongly supported division between the Nearctic and Palaearctic populations, which differentiated during the Pleistocene (~2.5 Mya). Populations from the Palaearctic region mostly diverged ~0.5 Mya. The topology of the tree is congruent with the haplotype network (Fig 4B).

### Species distribution modelling (SDM)

#### Boloria eunomia

**Fig 5:**
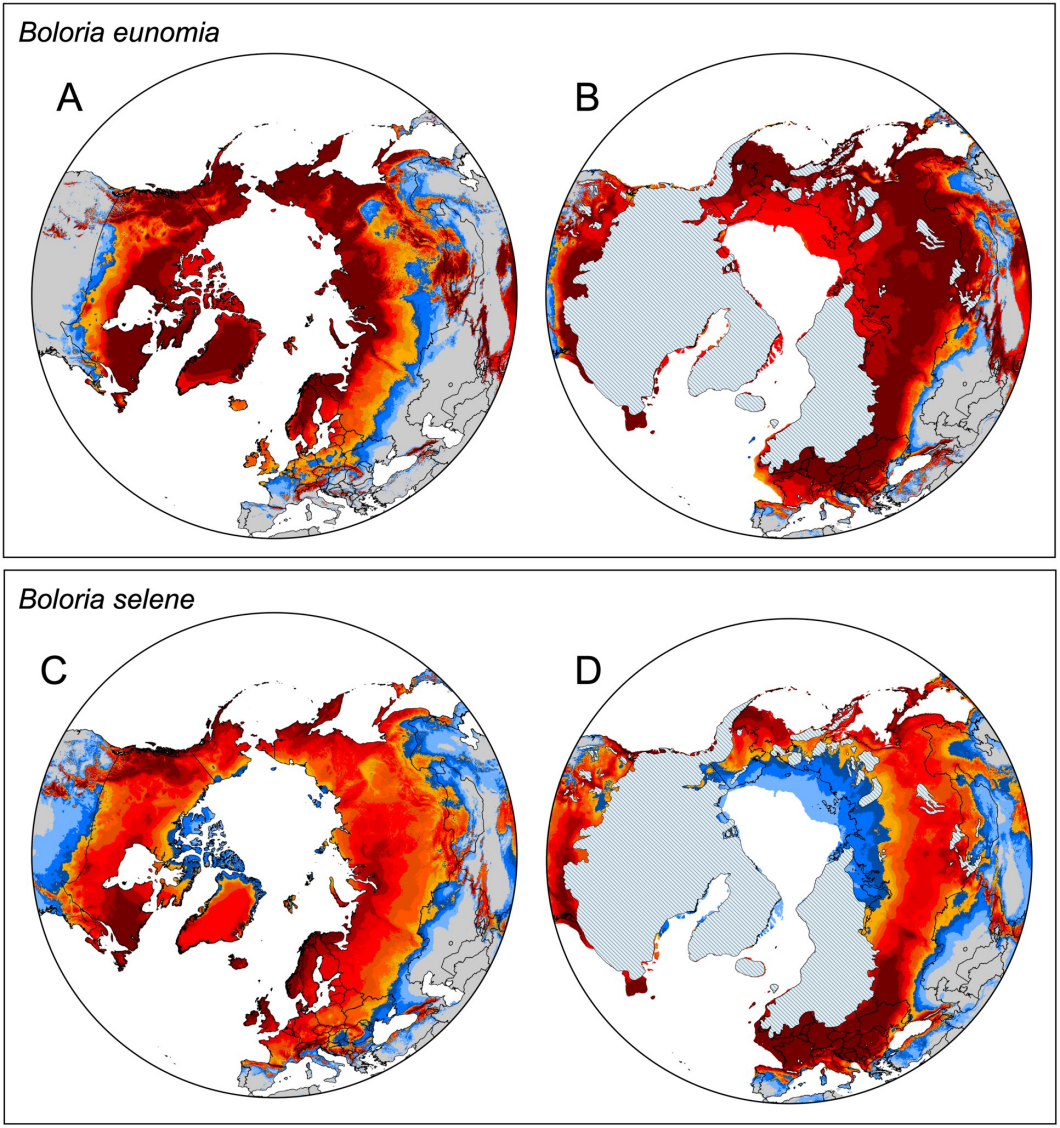
Species distribution models. SDMs showing the climatically suitable areas for *Boloria eunomia* at (A) present conditions and (B) LGM (about 22,000 years ago) and *Boloria selene* (C) present conditions, (D) LGM. The blue colours show the suitability from 0.14 for *B*. *eunomia* and 0.064 for *B*. *selene* (the average minimal training presence threshold, including the least suitable training record) and the red colours from 0.347 for *B*. *eunomia* and 0.338 for *B*. *selene* (the average ten percentile training presence threshold, suitability of 90% of the training data). The LGM glacial sheet was redrawn from Ehlers *et al*. [66]; the drawing is not identical to the original shape file and therefore, it is only for illustrative purposes.

The best RM was according to AICc = 4.5 and the most parsimonious function of FC was a hinge feature (H) (S2 Table). The average area under the curve (AUC) over the ten replicate runs was 0.870 (± 0.029 SD), indicating a good fit for the model. The selected most important BIOCLIM variables after the jackknife procedure, and the respective percentages of explained variation, were as follows: BIO5 (maximum temperature of warmest month), 95%; BIO7 (temperature annual range), 3.6%; BIO18 (precipitation of warmest quarter), 1.4%. Based on the selection of variables, the distribution of *B*. *eunomia* appears to be controlled mostly by temperature. The potential present distribution (Fig 5A) of the species covers large parts of boreal and Arctic Eurasia and North America, whereas the southern parts of the distribution are modelled as a series of isolated refugia. Some of them are actually occupied by the species (for instance the Alps, Pyrenees, Stara Planina, Caucasus, Altai, Rocky Mts.), whereas some are unoccupied (e.g., Iceland, UK, Sudeten Mts., Carpathians, Pamir Mts.). During the LGM, the model showed widespread distribution across the entire species distribution range reaching further south than in present (Fig 5B).

#### Boloria selene

The selected RM was 4 and the FC was a linear, quadratic, hinge feature (LQH) (S2 Table). The average AUC was 0.793 (± 0.034 SD). The selected BIOCLIM variables, and the respective percentages of explained variation, were: BIO10 (mean temperature of warmest quarter), 50%; BIO18 (precipitation of warmest quarter), 30%; BIO5 (maximum temperature of warmest month), 10.8%; BIO15 (precipitation seasonality), 8.3% and BIO2 (mean diurnal range), 0.9%. The distribution of *B*. *selene* also appears to be controlled by temperature. The potential present distribution of *B*. *selene* is similar to *B*. *eunomia*, but less isolated in southern areas. For instance, occupied isolated distribution is shown for Iberian chain or Caucasus, unoccupied but climatically suitable areas for Iceland or in Central Asian mountains (Fig 5C). The LGM model again showed continuous distribution all across the Holarctic region but with retreat from the northernmost parts of the actual species distribution towards south (Fig 5D).

## Discussion

Based on mitochondrial and nuclear markers and species distribution models, we assessed the biogeographic patterns of two Holarctic butterfly species *Boloria eunomia* and *Boloria selene*, providing a comprehensive perspective on the processes affecting these butterflies through the Quaternary.

### Palaearctic origin and colonization of America

**Fig 6:**
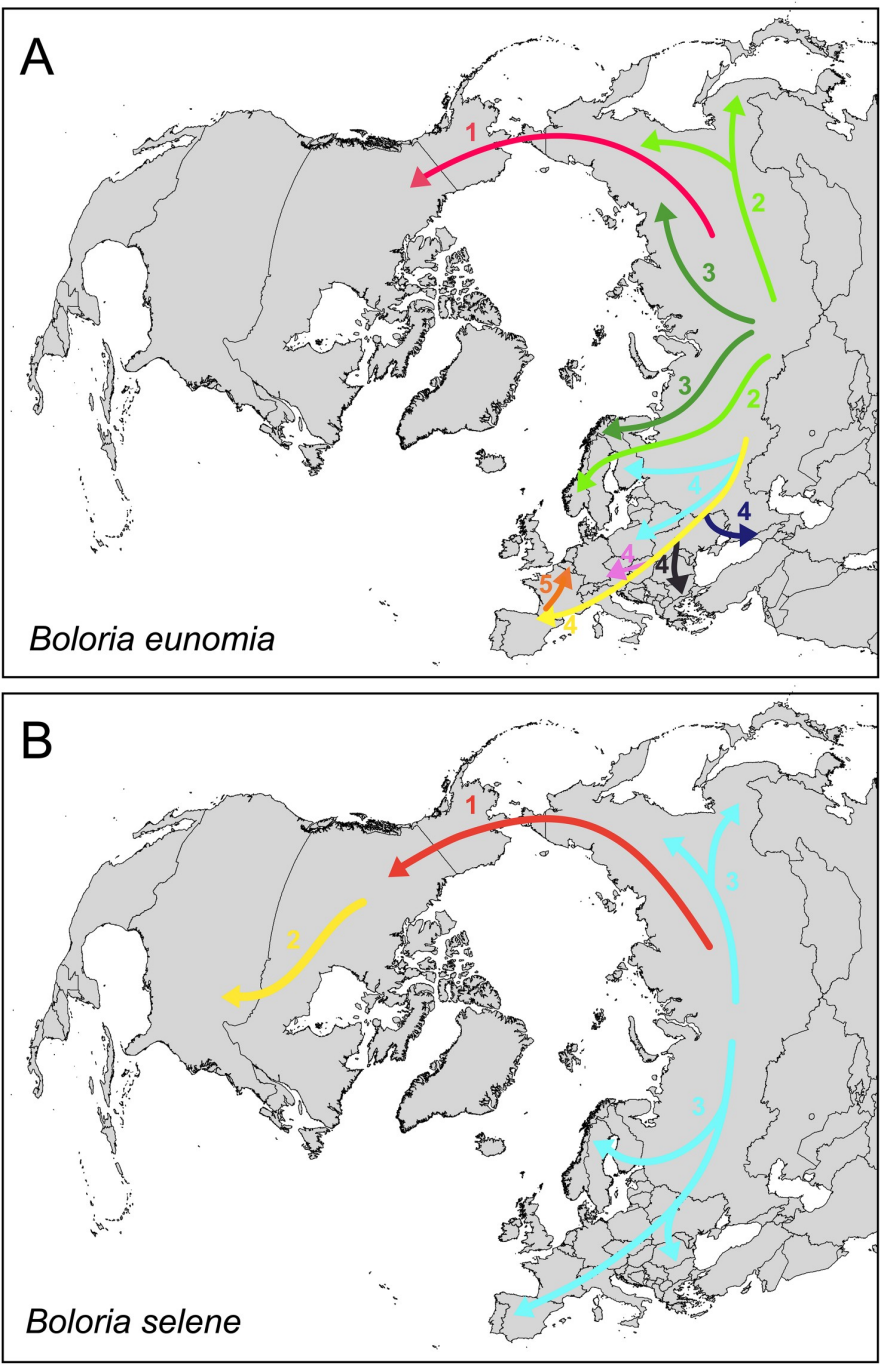
Hypothetical scenarios of colonization pathways for *Boloria eunomia* (A) and *Boloria selene* (B). Different colours and numbers indicate movement of each haplotype group illustrated in Fig 1 (#1 NA; #2 CS-SIB2; #3 NS-SIB1; #4 A1-CZ, A2, CA-PY, EE-UR, CAU; #5 AR-NI) and Fig 4 (#1 NA; #2 W; #3 E).

Central Asia is believed to be the evolutionary cradle for many cold-adapted butterflies such as the genera *Parnassius* Latreille, 1804 [67], *Oeneis* Hübner, [1819] [68], *Erebia* Dalman, 1816 [69], *Coenonympha* Hübner, [1819] [70,71], including *Boloria* Moore, 1900 [49,72]. Our study species, *B*. *eunomia* and *B*. *selene* probably also originated in the Palaearctic region [49]. In the case of *B*. *eunomia*, our results showed the highest genetic diversity within Siberia, supporting the Asian origin (Fig 1B and Table 2). Consequently, both butterflies expanded from Siberia across the other parts of the Holarctic region (Fig 6).

*Boloria selene* most likely colonised North America around 2.5 Mya (Fig 4C) [49] through the second Beringian land bridge where a continuous forest belt connected both continents which might have allowed the dispersal of boreal taxa [73]. This route appears to have been used by other butterflies, with contrasting climatic and habitat affinities, for instance the temperate-arboreal genus *Limenitis* Fabricius, 1807 [74], the warm grasslands generalist *Junonia* Hübner, [1819] [75], the cold-adapted *Oeneis* [66] and other members of the Satyrinae subfamily [69,76,77]. During the further Quaternary cooling and aridification, the third Beringian land bridge re-emerged [73], enabling the colonisation of the Nearctic region by the more cold-tolerant *B*. *eunomia* around 1.6 Mya (Fig 1C) [49]. Both studied butterflies colonised the Nearctic region only once, long before LGM, they share the significant genetic divergence between populations from Eurasia and North America (Figs 1–4), and were probably isolated during multiple upper Quaternary glacial cycles. The dry climate and tundra habitat might have acted as a barrier to species like *B*. *eunomia* and *B*. *selene*, preferring more humid or mesic conditions, respectively. Similar differentiation was observed in several other Lepidoptera (cf. [49,78]) and small mammal species [79–81].

### Biogeography in North America

Within North America, both species differ in their genetic structures. Our results imply only one glacial refugium for *B*. *eunomia*. Star-like pattern in haplotype network (Fig 1B), low genetic diversity across vast area of the Nearctic and non-significant neutrality tests (Table 2) suggest recent population expansion, probably from never glaciated Alaska [82] or southern Canada and northern USA, south of the Laurentide Ice Sheet. Unfortunately, without samples from southern parts of Rocky Mts., we are unable to choose between these alternatives.

In comparison with *B*. *eunomia*, *B*. *selene* colonised North America much earlier and is characterised by three different lineages within the region (Figs 2C and 4B), implying glacial survival not only in Alaska but also south of the Laurentide ice-sheet in the so-called ‘Driftless Region’ and in Rocky Mts. Previous phylogeographic and palaeontological studies suggest that the ‘Driftless Region’ (south-western Wisconsin, south-eastern Minnesota and north-eastern Iowa) served as a refugium for many other plant and animal species [83–90]. This is supported by the occurrence of unique haplotypes in the Wisconsin *B*. *selene* population (Fig 4B) and the highest genetic diversity within the species´ Nearctic range (Table 2). The third *B*. *selene* lineage is formed by two private haplotypes from Alberta (Fig 4B) suggesting another refugium and/or physical barriers within Rocky Mts. The split between haplotypes from Alberta and British Columbia was also observed in two moth species *Malacosoma disstria* (Hübner, 1820) and *M*. *californica* (Packard, 1864) [91]. All these possible LGM refugia are in concordance with the SDM models (Fig 5B and 5D) showing suitable areas within Beringia, north-west Rocky Mts. and south of the Laurentide ice-shield.

### *Boloria eunomia*—Palaearctic phylogeography

Contrary to the findings of Yakovlev et al. [92], our results confirm a high degree of phylogeographic structure within the *Boloria eunomia* Eurasian range. A high within-range genetic differentiation can be observed in other habitat specialist butterflies, for instance the semi-migratory species of grasslands *Hesperia comma* (Linnaeus, 1758) [93], the open-woodland specialist *Parnassius mnemosyne* (Linnaeus, 1758) [94], the polycentric dry grassland species *Melitaea cinxia* (Linnaeus, 1758) [95] or the steppic *Proterebia afra* (Fabricius, 1787) [96]. We identified ten population groups of *B*. *eunomia* (Fig 2A) within the Palaearctic region, mostly differentiating around 0.5 Mya (Figs 1C and 3A), corresponding with the onset of the long 100 kya glacial cycles of the Middle and Late Pleistocene. Only two lineages spread over the vast area of Siberia, leaving the highest geographic diversity to Europe, indicating different species´ histories across continents.

#### Dynamics in Siberia, Fennoscandia and Eastern Europe

Two extensive lineages exist in Siberia (NS-SIB1, SIB2; Figs 1A–1C and 2A), harbouring the highest haplotype diversity within the species (Table 2), possibly as a result of an interconnected network of populations during both glacials and interglacials. Siberia endured much less severe glaciations compared to Europe [97]. Moreover, this scenario is supported by the wide potential distribution during the LGM (Fig 5B), and the lack of geography-dependent genetic differentiation. Also, the archaeobotanical data and pollen records confirm high abundances of *B*. *eunomia* larval food plants (*Polygonum bistorta* and *P*. *viviparum*) in the European tundra about 15 kya (e.g. [98]). Such pattern had been already reported in another boreo-montane butterfly *Lycaena helle* [13].

As shown in other boreo-montane species [14,99–101], the two eastern lineages are responsible for post-glacial (re)colonisation of Fennoscandia (Figs 1 and 6A). The third lineage (EE-UR) reaching Scandinavia encompasses large parts of Eastern Europe with affinities to Ural Mts. and the Balkans (Stara Planina Mts.) (Figs 1A–1C and 2A). The proximity of Bulgarian and Polish populations may suggest a hypothetical existence of now extinct Carpathian populations of this species.

Not only Scandinavia but also Poland might have acted as contact zone for various lineages of *B*. *eunomia* as hypothesised by Krzywicki [102]. Besides of the EE-UR lineage, it also consists of an independent Polish clade (PL) and it shares haplotypes with NS-SIB1 lineage (Figs 1 and 2A and 2B and Fig B in S1 Fig). Similar phenomenon was observed for other butterflies [94,103,104]. It is also worth noting that in Poland two morphological forms of the butterfly showing distinct ecological characteristics co-occurs [105]. Detailed studies on relationships between those populations would be important for understanding of evolution of host races and validity of subspecies division suggested e.g. by Tolman & Lewington [32].

#### Diversification among European mountain ranges

We detected strong differentiation among most European mountain ranges (Cantabrian, Pyrenees, Ardennes, Alps, Bohemian Forest, Stara Planina and Caucasus Mts.) indicating restricted gene flow and long-term isolation of European populations, sufficient to fix population-specific haplotypes (Fig 1B). This genetic structuring contradicts our SDMs showing species’ widespread distribution during the LGM. However, more severe glaciations in the past (Elsterian/Mindel and Saalian/Riss) could have forced even cold-adapted organisms as *B*. *eunomia* to retreat within several refugia [106]. Mountains offer a heterogeneous topography with favourable microhabitats allowing the permanent persistence of various species over several glacial cycles by altitudinal shifts. At the same time, mountain ranges acted as barrier for the gene flow among populations, resulting in high geographic differentiation. This is the common pattern in the cold-adapted species in general [107] including boreo-montane butterflies [13,14].

Two *B*. *eunomia* lineages evolved within the Alps (A1-CZ, A2; Figs 1B and 2A), as a result of separation during the range contractions. The eastern lineage A1-CZ colonized the Bohemian Forest (Czech Republic), where the species persisted continuously at least since the end of the last glacial period [25]. Within south-western Europe, the SAMOVA analysis (Fig 2A) recognised two more closely related lineages (Fig 1B and 1C), one including Belgian and French populations (AR-NI), the other covering the Iberian peninsula (CA-PY), confirming the morphometric differentiation of these populations and the lower genetic variation of Ardennes populations compared to Pyrenees’ ones [108]. Furthermore, the haplotype network and Bayesian tree (Fig 1B and 1C) detected a differentiation between Cantabrian Mts. and Pyrenees, another example of population separation among different mountain ranges, reported for many plant and animal species [11,109–112]. Population in Caucasus Mts. (CAU) seems to be of the European origin as both mtDNA (Fig 1C) and nDNA (Fig 3D and 3E) suggest affinity to the western rather than the eastern samples. High haplotype diversity (Fig 1B and Table 2) and SDMs (Fig 5A and 5B), showing relative isolation of Caucasus during both present and LGM periods, also supports long-lasting persistence and isolation of the Caucasian population.

### *Boloria selene*—Evolutionary history within Eurasia

Within the Palaearctic region, both species are characterised by opposite genetic patterns to the ones in the Nearctic. Populations of *Boloria selene* are homogenously distributed and interconnected throughout the large parts of its Eurasian distribution range, reflected by one dominant haplotype (Fig 4B). Shallow mitogenetic/geographic structure in Lepidoptera across large species ranges similar to *B*. *selene* has been reported for several other butterfly species: two Holarctic—*Parnassius phoebus* (Fabricius, 1793) [113] and *Parasemia plantaginis* (Linnaeus, 1758) [114]; and three Palaearctic species–*Polygonia c-album* (Linnaeus, 1758) [115], *Lopinga achine* (Scopoli, 1763) [116] or *Aglais urticae* (Linnaeus, 1758) [117]. High haplotype diversity, significant negative neutrality tests (Table 2), the wide geographic distribution of common haplotypes, presence of many private haplotypes, and at the same time the lack of geographic structure (Fig 4A and 4B) suggest ongoing gene flow among *B*. *selene* populations with a recent population expansion (Fig 6B). This is in agreement with mark-recapture data, as the species easily disperse and demonstrates a low site fidelity [118]. On the other hand, the dispersal of *B*. *eunomia* is restricted, especially in a fragmented landscape [119]. SDMs (Fig 5C and 5D) indicated widespread distribution in both present and LGM, showing the interconnection of populations through the Quaternary.

## Conclusions

Despite the considerable overlap in their distribution, the genetic patterns in the two *Boloria* species notably differed. *B*. *eunomia* is characterised by a high genetic structuring across Europe, but panmixia in Asia and a lack of genetic differentiation across the Nearctic region. In contrast, *B*. *selene* is genetically strongly differentiated across North America and shows gene flow and thus low geographic differentiation across Eurasia. Generalist taxa such as the butterfly *B*. *selene* tend to have high genetic diversities and a low genetic differentiation among local populations. On the other hand, habitat specialists such as the butterfly species *B*. *eunomia* are affected from population isolation, which causes strong genetic differentiation [120]. This coherence becomes approved by the genetic signature obtained for the Palaearctic range for *B*. *selene* and for the European range for *B*. *eunomia*. Therefore, global evolutionary history of boreo-montane and boreo-temperate butterflies cannot be explained by the single biogeographic scenario proposed for cold-adapted species by Schmitt [8] but rather by combination of them, emphasising the different species´ histories in distinct parts of the distribution range. We highlight the importance of studying the genetic diversity within the entire species distribution ranges, which helps us to understand how the evolutionary history of organisms influenced their present distribution, and, thus to prioritize areas of conservation.

## Supporting information

S1 Fig Detailed maps of haplotype diversity of *Boloria eunomia* within Europe based on COI. Two lineages occurring in the Alps and the Czech Republic (A). Haplotype diversity in north-eastern Europe showing the contact zones in Scandinavia and Poland (B).
(TIF)

S2 Fig Graphs from SAMOVA analyses. *Boloria eunomia* cytochrome *c* oxidase subunit I (COI; 11 populations) (A); and *arginine kinase* gene (ArgKin; 3 populations) (B); *Boloria selene cytochrome c oxidase subunit* I (COI; 6 populations) (C). K, number of clusters; Φ_CT_, fixation index.
(TIF)

S1 Table Overview of samples of both *Boloria eunomia* and *Boloria selene*. Specimens localities; coordinates; COI, ArgKin and Wingless haplotypes, SAMOVA groups and GenBank accession numbers.
(XLSX)

S2 Table MaxEnt. Comparisons of (A) Regularization Multiplier (RM) parameter and (B) different feature classes (FC) of MaxEnt models by ENMeval R package using AICc, used for calculations of Spatial Distribution Models of *Boloria eunomia* and *Boloria selene*. The lowest AICc values indicate the best models. (C) Relative gain of the MaxEnt model after removing each of the variables. Only variables with positive gain larger than 0.01 were used.
(XLSX)
