# Supplementary figures and images for: Cross-continental phylogeography of two Holarctic Nymphalid butterflies, Boloria eunomia and Boloria selene
Source: PLoS One. 2019 Mar 26;14(3):e0214483. doi: 10.1371/journal.pone.0214483 (PMC6435151; doi:10.1371/journal.pone.0214483)

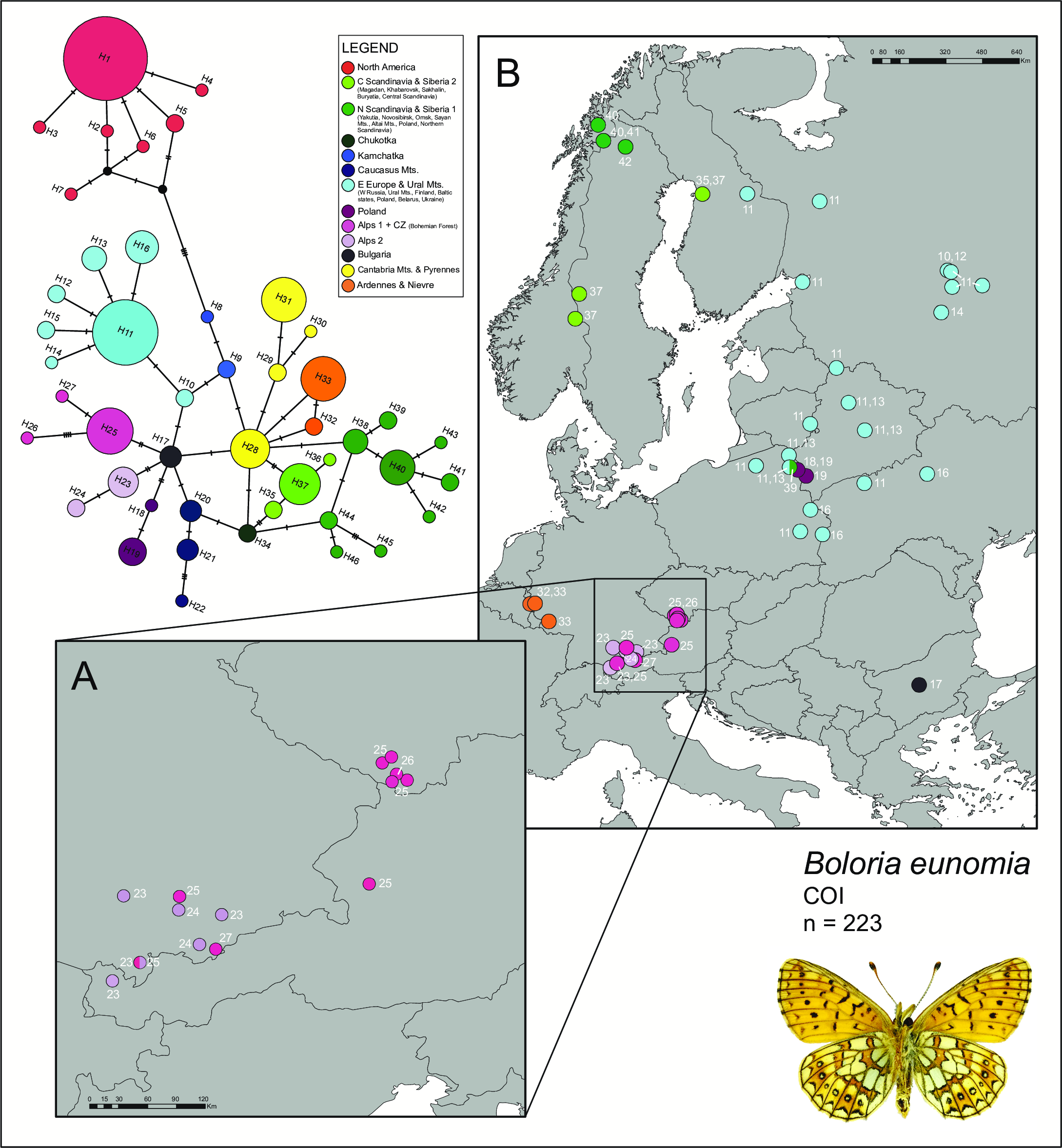

Supplement: S1 Fig — Two lineages occurring in the Alps and the Czech Republic (A). Haplotype diversity in north-eastern Europe showing the contact zones in Scandinavia and Poland (B). (TIF) [file pone.0214483.s001.tif]

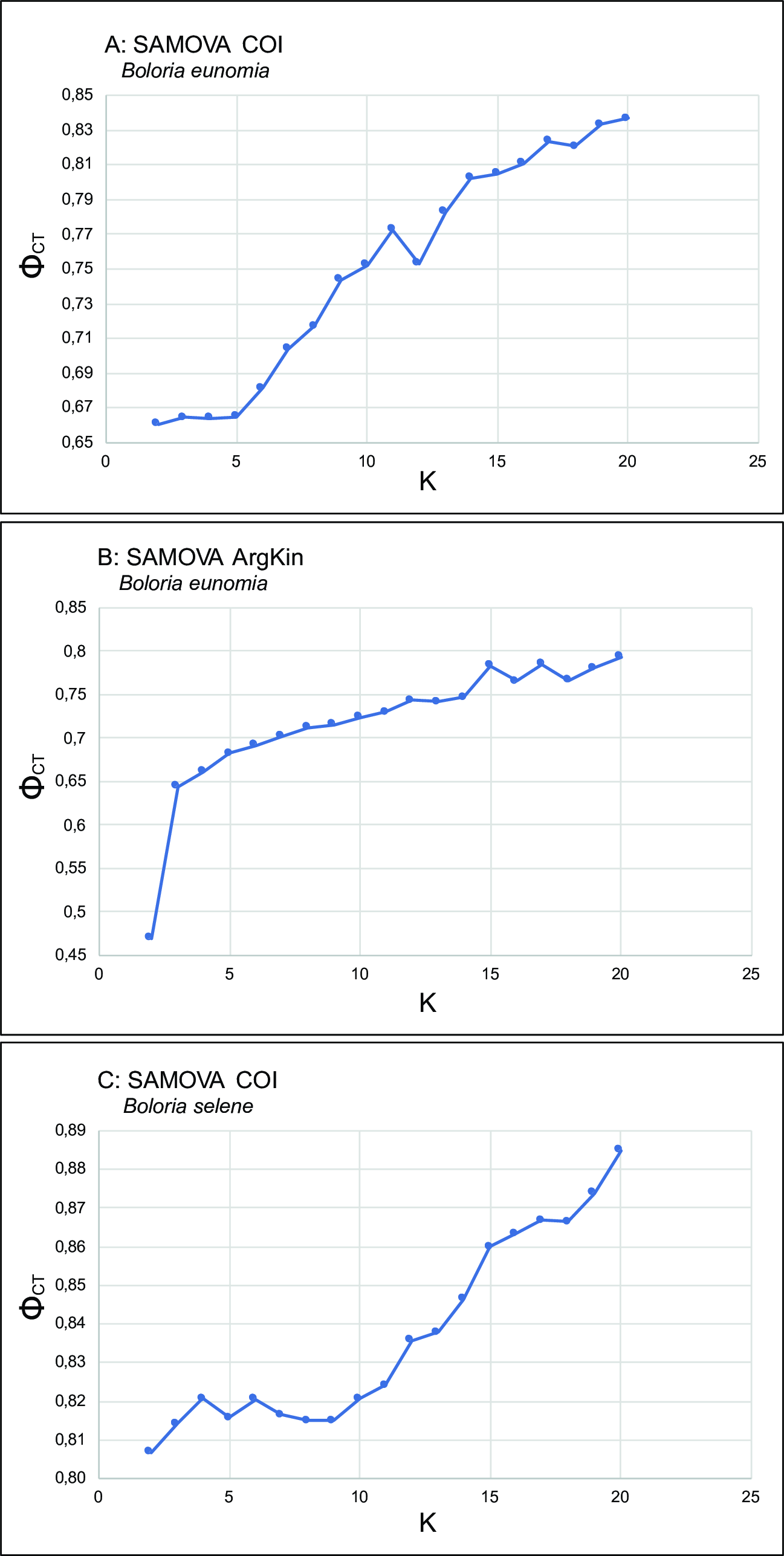

Supplement: S2 Fig — Boloria eunomia cytochrome c oxidase subunit I (COI; 11 populations) (A); and arginine kinase gene (ArgKin; 3 populations) (B); Boloria selene cytochrome c oxidase subunit I (COI; 6 populations) (C). K, number of clusters; ΦCT, fixation index. (TIF) [file pone.0214483.s002.tif]
